# Supplementary material for: Scaling of thermal tolerance with body mass and genome size in ectotherms: a comparison between water- and air-breathers
Source: Philos Trans R Soc Lond B Biol Sci. 2019 Jun 17;374(1778):20190035. doi: 10.1098/rstb.2019.0035 (PMC6606457; doi:10.1098/rstb.2019.0035)
Supplement: References for Raw data [file rstb20190035supp4.docx]

**References**

1. Katzenberger M, Hammond J, Tejedo M, Relyea R. 2018 Source of environmental data and warming tolerance estimation in six species of North American larval anurans. *J. Therm. Biol.* **76**, 171–178.

2. von May R, Catenazzi A, Corl A, Santa-Cruz R, Carnaval AC, Moritz C. 2017 Divergence of thermal physiological traits in terrestrial breeding frogs along a tropical elevational gradient. *Ecol. Evol.* **7**, 3257–3267.

3. Gutiérrez-Pesquera LM, Tejedo M, Olalla-Tárraga M, Duarte H, Nicieza A, Solé M. 2016 Testing the climate variability hypothesis in thermal tolerance limits of tropical and temperate tadpoles. *J. Biogeogr.* **43**, 1166–1178.

4. Storlie C, Merino-Viteri A, Phillips B, VanDerWal J, Welbergen J, Williams S. 2014 Stepping inside the niche: microclimate data are critical for accurate assessment of species’ vulnerability to climate change. *Biol. Lett.* **10**, 20140576.

5. Markle TM, Kozak KH. 2018 Low acclimation capacity of narrow-ranging thermal specialists exposes susceptibility to global climate change. *Ecol. Evol.*

6. Berkhouse CS, Fries JN. 1995 Critical thermal maxima of juvenile and adult San Marcos salamanders (*Eurycea nana*). *Southwest. Nat.* **40**, 430–434.

7. Davies SJ, McGeoch MA, Clusella-Trullas S. 2015 Plasticity of thermal tolerance and metabolism but not water loss in an invasive reed frog. *Comp. Biochem. Physiol. A. Mol. Integr. Physiol.* **189**, 11–20.

8. Barria AM, Bacigalupe LD. 2017 Intraspecific geographic variation in thermal limits and acclimatory capacity in a wide distributed endemic frog. *J. Therm. Biol.* **69**, 254–260.

9. Enríquez-Urzelai U. 2018 Integrating ecophysiology, complex life histories, and niche models to forecast the impact of climate change: a mechanistic assessment of the vulnerability of European common frogs (*Rana temporaria*). PhD Thesis, Oviedo, Spain.

10. Holzman N, McManus JJ. 1973 Effects of acclimation on metabolic rate and thermal tolerance in the carpenter frog, *Rana vergatipes*. *Comp. Biochem. Physiol. A Physiol.* **45**, 833–842.

11. Alveal-Riquelme N, Díaz-Páez H, Ortiz JC. 2016 Thermal tolerance in the Andean toad *Rhinella spinulosa* (Anura: Bufonidae) at three sites located along a latitudinal gradient in Chile. *J. Therm. Biol.* **60**, 237–245.

12. Bury RB. 2008 Low thermal tolerances of stream amphibians in the Pacific Northwest: Implications for riparian and forest management. *Appl. Herpetol.* **5**, 63–74.

13. Gvoždík L, Puky M, Šugerkova M. 2007 Acclimation is beneficial at extreme test temperatures in the Danube crested newt, *Triturus dobrogicus* (Caudata, Salamandridae). *Biol. J. Linn. Soc.* **90**, 627–636.

14. van den Berg FT, Thompson MB, Hochuli DF. 2015 When hot rocks get hotter: behavior and acclimatization mitigate exposure to extreme temperatures in a spider. *Ecosphere* **6**, 1–17.

15. Janion-Scheepers C, Phillips L, Sgrò CM, Duffy GA, Hallas R, Chown SL. 2017 Basal resistance enhances warming tolerance of alien over indigenous species across latitude. *Proc. Natl. Acad. Sci.* , 201715598.

16. Klok CJ, Sinclair BJ, Chown SL. 2004 Upper thermal tolerance and oxygen limitation in terrestrial arthropods. *J. Exp. Biol.* **207**, 2361–2370.

17. Verberk WCEP, Leuven RS, van der Velde G, Gabel F. 2018 Thermal limits in native and alien freshwater peracarid Crustacea: The role of habitat use and oxygen limitation. *Funct. Ecol.* **32**, 926–936.

18. Ern R, Phuong NT, Madsen PT, Wang T, Bayley M, others. 2015 Some like it hot: thermal tolerance and oxygen supply capacity in two eurythermal crustaceans. *Sci. Rep.* **5**, 10743.

19. Noyola-Regil J, Mascaro M, Diaz F, Re AD, Sánchez-Zamora A, Caamal-Monsreal C, Rosas C. 2015 Thermal biology of prey (*Melongena corona bispinosa*, *Strombus pugilis*, *Callinectes similis*, *Libinia dubia*) and predators (*Ocyurus chrysurus*, *Centropomus undecimalis*) of *Octopus maya* from the Yucatan Peninsula. *J. Therm. Biol.* **53**, 151–161.

20. Cuculescu M, Hyde D, Bowler K. 1998 Thermal tolerance of two species of marine crab, *Cancer pagurus* and *Carcinus maenas*. *J. Therm. Biol.* **23**, 107–110.

21. Magozzi S, Calosi P. 2015 Integrating metabolic performance, thermal tolerance, and plasticity enables for more accurate predictions on species vulnerability to acute and chronic effects of global warming. *Glob. Change Biol.* **21**, 181–194.

22. Geerts AN *et al.* 2015 Rapid evolution of thermal tolerance in the water flea *Daphnia*. *Nat. Clim. Change* **5**, 665.

23. Wijnhoven S, Van Riel MC, van der Velde G. 2003 Exotic and indigenous freshwater gammarid species: physiological tolerance to water temperature in relation to ionic content of the water. *Aquat. Ecol.* **37**, 151–158.

24. Cumillaf JP, Blanc J, Paschke K, Gebauer P, Díaz F, Re D, Chimal ME, Vásquez J, Rosas C. 2016 Thermal biology of the sub-polar–temperate estuarine crab *Hemigrapsus crenulatus* (Crustacea: Decapoda: Varunidae). *Biol. Open* , bio–013516.

25. McGaw IJ. 2003 Behavioral thermoregulation in *Hemigrapsus nudus*, the amphibious purple shore crab. *Biol. Bull.* **204**, 38–49.

26. Paschke K, Cumillaf JP, Chimal ME, Díaz F, Gebauer P, Rosas C. 2013 Relationship between age and thermoregulatory behaviour of *Lithodes santolla* (Molina, 1782)(Decapoda, Lithodidae) juveniles. *J. Exp. Mar. Biol. Ecol.* **448**, 141–145.

27. Kumlu M, Türkmen S, Kumlu M. 2010 Thermal tolerance of *Litopenaeus vannamei* (Crustacea: Penaeidae) acclimated to four temperatures. *J. Therm. Biol.* **35**, 305–308.

28. Dı́az F, Sierra E, Re AD, Rodrı́guez L. 2002 Behavioural thermoregulation and critical thermal limits of *Macrobrachium acanthurus* (Wiegman). *J. Therm. Biol.* **27**, 423–428.

29. Selvakumar S, Geraldine P. 2005 Heat shock protein induction in the freshwater prawn *Macrobrachium malcolmsonii*: acclimation-influenced variations in the induction temperatures for Hsp70. *Comp. Biochem. Physiol. A. Mol. Integr. Physiol.* **140**, 209–215.

30. Dı́az F, Uribe ES, Ramirez LFB, Mora AG. 1998 Critical thermal maxima and minima of *Macrobrachium rosenbergii* (Decapoda: Palaemonidae). *J. Therm. Biol.* **23**, 381–385.

31. Hernández M, Ramirez LFB, Díaz F. 1996 Critical thermal maximum of *Macrobrachium tenellum*. *J. Therm. Biol.* **21**, 139–143.

32. Morritt D, Ingólfsson A. 2000 Upper thermal tolerances of the beachflea *Orchestia gammarellus* (Pallas)(Crustacea: Amphipoda: Talitridae) associated with hot springs in Iceland. *J. Exp. Mar. Biol. Ecol.* **255**, 215–227.

33. Claussen DL. 1980 Thermal acclimation in the crayfish, *Orconectes rusticus* and *O. virilis*. *Comp. Biochem. Physiol. A Physiol.* **66**, 377–384.

34. Peck LS, Morley SA, Clark MS. 2010 Poor acclimation capacities in Antarctic marine ectotherms. *Mar. Biol.* **157**, 2051–2059.

35. Clark TD, Roche DG, Binning SA, Speers-Roesch B, Sundin J. 2017 Maximum thermal limits of coral reef damselfishes are size dependent and resilient to near-future ocean acidification. *J. Exp. Biol.* **220**, 3519–3526.

36. Stevens MM, Jackson S, Bester SA, Terblanche JS, Chown SL. 2010 Oxygen limitation and thermal tolerance in two terrestrial arthropod species. *J. Exp. Biol.* **213**, 2209–2218.

37. Qari S, Aljarari R. 2014 The effect of season and acclimation on the heat and cold tolerance of the Red Sea Crab, *Portunus pelagicus*. *Life Sci J* **11**, 145–148.

38. Payette AL, McGaw IJ. 2003 Thermoregulatory behavior of the crayfish *Procambarus clarki* in a burrow environment. *Comp. Biochem. Physiol. A. Mol. Integr. Physiol.* **136**, 539–556.

39. Darnell MZ, Nicholson HS, Munguia P. 2015 Thermal ecology of the fiddler crab Uca panacea: thermal constraints and organismal responses. *J. Therm. Biol.* **52**, 157–165.

40. Darnell MZ, Darnell KM. 2018 Geographic variation in thermal tolerance and morphology in a fiddler crab sister-species pair. *Mar. Biol.* **165**, 26.

41. Tsuchida S. 1995 The relationship between upper temperature tolerance and final preferendum of Japanese marine fish. *J. Therm. Biol.* **20**, 35–41.

42. Ziegeweid JR, Jennings CA, Peterson DL. 2008 Thermal maxima for juvenile shortnose sturgeon acclimated to different temperatures. *Environ. Biol. Fishes* **82**, 299–307.

43. Verhille CE, Lee S, Todgham AE, Cocherell DE, Hung SS, Fangue NA. 2016 Effects of nutritional deprivation on juvenile green sturgeon growth and thermal tolerance. *Environ. Biol. Fishes* **99**, 145–159.

44. Campos DF, Val AL, Almeida-Val VMF. 2018 The influence of lifestyle and swimming behavior on metabolic rate and thermal tolerance of twelve Amazon forest stream fish species. *J. Therm. Biol.* **72**, 148–154.

45. Murchie KJ, Cooke SJ, Danylchuk AJ, Danylchuk SE, Goldberg TL, Suski CD, Philipp DP. 2011 Thermal biology of bonefish (*Albula vulpes*) in Bahamian coastal waters and tidal creeks: an integrated laboratory and field study. *J. Therm. Biol.* **36**, 38–48.

46. Wood CM *et al.* 2016 Mammalian metabolic rates in the hottest fish on earth. *Sci. Rep*.**6**

47. Smale MA, Rabeni CF. 1995 Hypoxia and hypertermia tolerance of headwater stream fishes. *Trans Am Fish Soc* **124**, 698–710.

48. Carveth CJ, Widmer AM, Bonar SA. 2006 Comparison of upper thermal tolerances of native and nonnative fish species in Arizona. *Trans. Am. Fish. Soc.* **135**, 1433–1440.

49. Paschke K *et al.* 2018 Comparison of aerobic scope for metabolic activity in aquatic ectotherms with temperature related metabolic stimulation: a novel approach for aerobic power budget. *Front. Physiol.* **9**.

50. Sarma K, Pal AK, Ayyappan S, Das T, Manush SM, Debnath D, Baruah K. 2010 Acclimation of Anabas testudineus (Bloch) to three test temperatures influences thermal tolerance and oxygen consumption. *Fish Physiol. Biochem.* **36**, 85–90.

51. Simons M. 1984 *Species Specific Responses of Freshwater Organisms to Elevated Water Temperatures*. Waikato Valley Authority.

52. León-Palomino C, Flores-Mego J, Dionicio-Acedo J, Rosado-Salazar M, Flye-Sainte-Marie J, Aguirre-Velarde A. 2017 Preferencia y tolerancia térmica de juveniles de chita *Anisotremus scapularis* (Pisces: Haemulidae). *Rev. Biol. Mar. Oceanogr.* **52**, 581–589.

53. Jung HE. 2018 The relationship between thermal tolerance and hypoxia tolerance in Amazonian fishes. PhD Thesis, University of British Columbia.

54. Sharma NK, Akhtar MS, Pandey N, Singh R, Singh AK. 2015 Seasonal variation in thermal tolerance, oxygen consumption, antioxidative enzymes and non-specific immune indices of Indian hill trout, *Barilius bendelisis* (Hamilton, 1807) from central Himalaya, India. *J. Therm. Biol.* **52**, 166–176.

55. Eme J, Bennett WA. 2009 Critical thermal tolerance polygons of tropical marine fishes from Sulawesi, Indonesia. *J. Therm. Biol.* **34**, 220–225.

56. Rummer JL *et al.* 2009 Physiological tolerance to hyperthermia and hypoxia and effects on species richness and distribution of rockpool fishes of Loggerhead Key, Dry Tortugas National Park. *J. Exp. Mar. Biol. Ecol.* **371**, 155–162.

57. Chagnon N, Hlohowskyj I. 1989 Effects of phenol exposure on the thermal tolerance ability of the central stoneroller minnow. *Bull. Environ. Contam. Toxicol.* **42**, 614–619.

58. Das T, Pal AK, Chakraborty SK, Manush SM, Chatterjee N, Mukherjee SC. 2004 Thermal tolerance and oxygen consumption of Indian Major Carps acclimated to four temperatures. *J. Therm. Biol.* **29**, 157–163.

59. Deacon JE, Schumann PB, Stuenkel EL. 1987 Thermal tolerances and preferences of fishes of the Virgin River system (Utah, Arizona, Nevada). *Gt. Basin Nat.* , 538–546.

60. Beers JM, Sidell BD. 2011 Thermal tolerance of Antarctic notothenioid fishes correlates with level of circulating hemoglobin. *Physiol. Biochem. Zool.* **84**, 353–362.

61. Ern R, Johansen JL, Rummer JL, Esbaugh AJ. 2017 Effects of hypoxia and ocean acidification on the upper thermal niche boundaries of coral reef fishes. *Biol. Lett.* **13**, 20170135.

62. Mora C, Ospina A. 2001 Tolerance to high temperatures and potential impact of sea warming on reef fishes of Gorgona Island (tropical eastern Pacific). *Mar. Biol.* **139**, 765–769.

63. Walsh SJ, Haney DC, Timmerman CM. 1997 Variation in thermal tolerance and routine metabolism among spring-and stream dwelling freshwater sculpins (Teleostei: Cottidae) of the southeastern United States. *Ecol. Freshw. Fish* **6**, 84–94.

64. Bettoli PW, Neill WH, Kelsch SW. 1985 Temperature preference and heat resistance of grass carp, *Ctenopharyngodon idella* (Valenciennes), bighead carp, *Hypophthalmichthys nobilis* (Gray), and their F1 hybrid. *J. Fish Biol.* **27**, 239–247.

65. Ern R, Norin T, Garperl K, Esbaugh AJ. 2016 Oxygen dependence of upper thermal limits in fishes. *J. Exp. Biol.* **219**, 3376–3383. (doi:10.1242/jeb.143495)

66. Matthews W s J, Maness J. 1979 Critical Thermal Maxima, Oxygen Tolerances and Success of Cyprinid Fishes in a Southwestern River. *Am. Midl. Nat.* **102**, 374–377.

67. Hockett CT, Mundahl ND. 1989 Effects of Black Spot Disease on Thermal Tolerances and Condition Factors of Three Cyprinid Fishes. *J. Freshw. Ecol.* **5**, 67–72. (doi:10.1080/02705060.1989.9665214)

68. Heath AG, Turner BJ, Davis WP. 1993 Temperature preferences and tolerances of three fish species inhabiting hyperthermal ponds on mangrove islands. *Hydrobiologia* **259**, 47–55.

69. Bennett WA, Beitinger TL. 1997 Temperature tolerance of the sheepshead minnow, *Cyprinodon variegatus*. *Copeia* , 77–87.

70. Sidhu R, Anttila K, Farrell AP. 2014 Upper thermal tolerance of closely related *Danio* species. *J. Fish Biol.* **84**, 982–995.

71. Cortemeglia C, Beitinger TL. 2005 Temperature tolerances of wild-type and red transgenic zebra danios. *Trans. Am. Fish. Soc.* **134**, 1431–1437.

72. Dülger N, Kumlu M, Türkmen S, Ölçülü A, Eroldoğan OT, Yılmaz HA, Öçal N. 2012 Thermal tolerance of European Sea Bass (*Dicentrarchus labrax*) juveniles acclimated to three temperature levels. *J. Therm. Biol.* **37**, 79–82.

73. Fries JN, Gibson JR. 2010 Critical thermal maxima of captive-bred Devils River minnows (*Dionda diaboli*). *Southwest. Nat.* **55**, 544–551.

74. Madeira D, Narciso L, Cabral HN, Vinagre C. 2012 Thermal tolerance and potential impacts of climate change on coastal and estuarine organisms. *J. Sea Res.* **70**, 32–41.

75. Monirian J, Sutphin Z, Myrick C. 2010 Effects of holding temperature and handling stress on the upper thermal tolerance of threadfin shad *Dorosoma petenense*. *J. Fish Biol.* **76**, 1329–1342.

76. Di Santo V, Lobel PS. 2017 Body size and thermal tolerance in tropical gobies. *J. Exp. Mar. Biol. Ecol.* **487**, 11–17.

77. Lattuca ME, Boy CC, Vanella FA, Barrantes ME, Fernández DA. 2018 Thermal responses of three native fishes from estuarine areas of the Beagle Channel, and their implications for climate change. *Hydrobiologia* **808**, 235–249. (doi:10.1007/s10750-017-3424-8)

78. Hlohowskyj I, Wissing TE. 1985 Seasonal changes in the critical thermal maxima of fantail (*Etheostoma flabellare*), greenside (*Etheostoma blennioides*), and rainbow (*Etheostoma caeruleum*) darters. *Can. J. Zool.* **63**, 1629–1633.

79. Smith RK, Fausch KD. 1997 Thermal tolerance and vegetation preference of Arkansas darter and johnny darter from Colorado plains streams. *Trans. Am. Fish. Soc.* **126**, 676–686.

80. Rajaguru S. 2002 Critical thermal maximum of seven estuarine fishes. *J. Therm. Biol.* **27**, 125–128.

81. McArley TJ, Hickey AJ, Herbert NA. 2017 Chronic warm exposure impairs growth performance and reduces thermal safety margins in the common triplefin fish (*Forsterygion lapillum*). *J. Exp. Biol.* **220**, 3527–3535.

82. Fangue NA, Hofmeister M, Schulte PM. 2006 Intraspecific variation in thermal tolerance and heat shock protein gene expression in common killifish, *Fundulus heteroclitus*. *J. Exp. Biol.* **209**, 2859–2872.

83. Otto RG. 1974 The effects of acclimation to cyclic thermal regimes on heat tolerance of the western mosquitofish. *Trans. Am. Fish. Soc.* **103**, 331–335.

84. Feldmeth CR, Baskin JN. 1976 Thermal and respiratory studies with reference to temperature and oxygen tolerance for the unarmored stickleback *Gasterosteus aculeatus williamsoni* Hubbs. *Bull. South. Calif. Acad. Sci.* **75**, 127–131.

85. Bilyk KT, DeVries AL. 2011 Heat tolerance and its plasticity in Antarctic fishes. *Comp. Biochem. Physiol. A. Mol. Integr. Physiol.* **158**, 382–390.

86. Mascaró M, Amaral-Ruiz M, Huipe-Zamora I, Martínez-Moreno G, Simoes N, Rosas C. 2016 Thermal tolerance and phenotypic plasticity in juvenile *Hippocampus erectus* Perry, 1810: effect of acute and chronic exposure to contrasting temperatures. *J. Exp. Mar. Biol. Ecol.* **483**, 112–119.

87. Dalvi RS, Pal AK, Tiwari LR, Das T, Baruah K. 2009 Thermal tolerance and oxygen consumption rates of the catfish *Horabagrus brachysoma* (Günther) acclimated to different temperatures. *Aquaculture* **295**, 116–119.

88. Komoroske LM, Connon RE, Lindberg J, Cheng BS, Castillo G, Hasenbein M, Fangue NA. 2014 Ontogeny influences sensitivity to climate change stressors in an endangered fish. *Conserv. Physiol.* **2**.

89. Currie RJ, Bennett WA, Beitinger TL. 1998 Critical thermal minima and maxima of three freshwater game-fish species acclimated to constant temperatures. *Environ. Biol. Fishes* **51**, 187–200.

90. Billman EJ, Wagner EJ, Arndt RE, VanDyke E. 2008 Optimal temperatures for growth and upper thermal tolerance of juvenile northern leatherside chub. *West. North Am. Nat.* **68**, 463–475.

91. Dent L, Lutterschmidt WI. 2003 Comparative thermal physiology of two sympatric sunfishes (Centrarchidae: Perciformes) with a discussion of microhabitat utilization. *J. Therm. Biol.* **28**, 67–74.

92. Campbell CM, Davies PS. 1975 Thermal acclimation in the teleost, *Blennius pholis* (L). *Comp. Biochem. Physiol. A Physiol.* **52**, 147–151.

93. Thompson L, Fangue NA, Cech JJ, Cocherell DE, Kaufman RC. 2012 Juvenile and adult hardhead thermal tolerances and preferences: Temperature preference, critical thermal limits, active and resting metabolism, and blood-oxygen equilibria. Center for Aquatic Biology and Aquaculture Technical Report, University of California, Davis

94. Cross EE, Rawding RS. 2009 Acute thermal tolerance in the round goby, Apollonia melanostoma (*Neogobius melanostomus*). *J. Therm. Biol.* **34**, 85–92.

95. Hooper JK. 2008 The effect of temperature change on the New Zealand marine fish, *Notolabrus celidotus*. Master Thesis, University of Canterbury, New Zeland.

96. Lee RM, Rinne JN. 1980 Critical thermal maxima of five trout species in the southwestern United States. *Trans. Am. Fish. Soc.* **109**, 632–635.

97. Becker CD, Genoway RG. 1979 Evaluation of the critical thermal maximum for determining thermal tolerance of freshwater fish. *Environ. Biol. Fishes* **4**, 245.

98. Debnath D, Pal AK, Sahu NP, Baruah K, Yengkokpam S, Das T, Manush SM. 2006 Thermal tolerance and metabolic activity of yellowtail catfish *Pangasius pangasius* (Hamilton) advanced fingerlings with emphasis on their culture potential. *Aquaculture* **258**, 606–610.

99. Campos DF, Jesus TF, Kochhann D, Heinrichs-Caldas W, Coelho MM, Almeida-Val VMF. 2017 Metabolic rate and thermal tolerance in two congeneric Amazon fishes: *Paracheirodon axelrodi* Schultz, 1956 and *Paracheirodon simulans* Géry, 1963 (Characidae). *Hydrobiologia* **789**, 133–142.

100. Kim WS, Yoon S-J, Kim JM, Gil JW, Lee TW. 2005 Effects of temperature changes on the endogenous rhythm of oxygen consumption in the Japanese flounder *Paralichthys olivaceus*. *Fish. Sci.* **71**, 471–478.

101. Taylor JR, Cook MM, Kirkpatrick AL, Galleher SN, Eme J, Bennett WA. 2005 Thermal tactics of air-breathing and non air-breathing gobiids inhabiting mangrove tidepools on Pulau Hoga, Indonesia. *Copeia* **2005**, 886–893.

102. Di Santo V, Jordan HL, Cooper B, Currie RJ, Beitinger TL, Bennett WA. 2018 Thermal tolerance of the invasive red-bellied pacu and the risk of establishment in the United States. *J. Therm. Biol.* **74**, 110–115. (doi:10.1016/j.jtherbio.2018.03.015)

103. Messmer V, Pratchett MS, Hoey AS, Tobin AJ, Coker DJ, Cooke SJ, Clark TD. 2017 Global warming may disproportionately affect larger adults in a predatory coral reef fish. *Glob. Change Biol.* **23**, 2230–2240. (doi:10.1111/gcb.13552)

104. Hernández R. M, Bückle R. LF. 2002 Temperature tolerance polygon of *Poecilia sphenops* Valenciennes (Pisces: Poeciliidae). *J. Therm. Biol.* **27**, 1–5.

105. Chung KS. 2001 Critical thermal maxima and acclimation rate of the tropical guppy. Hidrobiologia *Poecilla reticulata*. *Hydrobiologia* **462**, 253–257. (doi:10.1023/A:1013158904036)

106. Young PS, Cech JJ. 1996 Environmental Tolerances and Requirements of Splittail. *Trans. Am. Fish. Soc.* **125**, 664–678.

107. Barrionuevo WR, Femandes M. 1995 Critical thermal maxima and minima for curimbatá, *Prochilodus scrofa* Steindachner, of two different sizes. *Aquac. Res.* **26**, 447–450. (doi:10.1111/j.1365-2109.1995.tb00934.x)

108. Chapman LJ, McDonnell LH. 2015 At the edge of the thermal window: effects of elevated temperature on the resting metabolism, hypoxia tolerance and upper critical thermal limit of a widespread African cichlid. *Conserv. Physiol.* **3**. (doi:10.1093/conphys/cov050)

109. Aikens C, Frost C, Henderson M, Kreisler S, Lopez E, Maynard N, White D. 2009 The Critical Thermal Maximum of Lionfish (*Pterois volitans*).

110. Pérez E, Dı́az F, Espina S. 2003 Thermoregulatory behavior and critical thermal limits of the angelfish *Pterophyllum scalare* (Lichtenstein) (Pisces: Cichlidae). *J. Therm. Biol.* **28**, 531–537. (doi:10.1016/S0306-4565(03)00055-X)

111. Kaya CM, Brussard PF, Cameron DG, Vyse ER. 1992 Biochemical Genetics and Thermal Tolerances of Kendall Warm Springs Dace (*Rhinichthys osculus thermalis*) and Green River Speckled Dace (R. o. yarrowi). *Copeia* **1992**, 528–535. (doi:10.2307/1446213)

112. Elliott JM, Elliott JA. 1995 The effect of the rate of temperature increase on the critical thermal maximum for parr of Atlantic salmon and brown trout. *J. Fish Biol.* **47**, 917–919. (doi:10.1111/j.1095-8649.1995.tb06014.x)

113. Madeira C, Mendonça V, Leal MC, Flores AAV, Cabral HN, Diniz MS, Vinagre C. 2017 Thermal stress, thermal safety margins and acclimation capacity in tropical shallow waters—An experimental approach testing multiple end-points in two common fish. *Ecol. Indic.* **81**, 146–158. (doi:10.1016/j.ecolind.2017.05.050)

114. Kita J, Tsuchida S, Setoguma T. 1996 Temperature preference and tolerance, and oxygen consumption of the marbled rockfish, *Sebastiscus marmoratus*. *Mar. Biol.* **125**, 467–471. (doi:10.1007/BF00353259)

115. McClanahan LL, Feldmeth CR, Jones J, Soltz DL. 1986 Energetics, Salinity and Temperature Tolerance in the Mohave Tui Chub, *Gila bicolor mohavensis*. *Copeia* **1986**, 45–52. (doi:10.2307/1444886)

116. Reyes I, Díaz F, Re AD, Pérez J. 2011 Behavioral thermoregulation, temperature tolerance and oxygen consumption in the Mexican bullseye puffer fish, *Sphoeroides annulatus* Jenyns (1842), acclimated to different temperatures. *J. Therm. Biol.* **36**, 200–205. (doi:10.1016/j.jtherbio.2011.03.003)

117. Lohr SC, Byorth PA, Kaya CM, Dwyer WP. 1996 High-Temperature Tolerances of Fluvial Arctic Grayling and Comparisons with Summer River Temperatures of the Big Hole River, Montana. *Trans. Am. Fish. Soc.* **125**, 933–939. (doi:10.1577/1548-8659(1996)125<0933:HTTOFA>2.3.CO;2)

118. Akhtar MS, Pal AK, Sahu NP, Ciji A, Mahanta PC. 2013 Thermal tolerance, oxygen consumption and haemato-biochemical variables of *Tor putitora* juveniles acclimated to five temperatures. *Fish Physiol. Biochem.* **39**, 1387–1398. (doi:10.1007/s10695-013-9793-7)

119. Matern SA. 2001 Using Temperature and Salinity Tolerances to Predict the Success of the Shimofuri Goby, a Recent Invader into California. *Trans. Am. Fish. Soc.* **130**, 592–599. (doi:10.1577/1548-8659(2001)130<0592:UTASTT>2.0.CO;2)

120. Prodocimo V, Freire CA. 2001 Critical thermal maxima and minima of the platyfish *Xiphophorus maculatus* Günther (Poecillidae, Cyprinodontiformes): a tropical species of ornamental freshwater fish. *Rev. Bras. Zool.* **18**, 97–106.

121. Zakhartsev MV, De Wachter B, Sartoris F-J, Pörtner H-O, Blust R. 2003 Thermal physiology of the common eelpout (Zoarces viviparus). *J. Comp. Physiol. B* **173**, 365–378.

122. Birkett AJ, Blackburn GA, Menéndez R. 2018 Linking species thermal tolerance to elevational range shifts in upland dung beetles. *Ecography* **41**, 1510–1519. (doi:10.1111/ecog.03458)

123. Verberk WCEP, Bilton DT. 2013 Respiratory control in aquatic insects dictates their vulnerability to global warming. *Biol. Lett.* **9**, 20130473.

124. Calosi P, Bilton DT, Spicer JI. 2008 Thermal tolerance, acclimatory capacity and vulnerability to global climate change. *Biol. Lett.* **4**, 99–102.

125. Hamblin April L., Youngsteadt Elsa, López-Uribe Margarita M., Frank Steven D. 2017 Physiological thermal limits predict differential responses of bees to urban heat-island effects. *Biol. Lett.* **13**, 20170125. (doi:10.1098/rsbl.2017.0125)

126. Ernst MR, Beitinger TL, Stewart KW. 1984 Critical Thermal Maxima of Nymphs of Three Plecoptera Species from an Ozark Foothill Stream. *Freshw. Invertebr. Biol.* **3**, 80–85. (doi:10.2307/1467096)

127. Nowrouzi S, Andersen AN, Bishop TR, Robson SK. 2018 Is thermal limitation the primary driver of elevational distributions? Not for montane rainforest ants in the Australian Wet Tropics. *Oecologia* , 1–10.

128. Verble-Pearson RM, Gifford ME, Yanoviak SP. 2015 Variation in thermal tolerance of North American ants. *J. Therm. Biol.* **48**, 65–68.

129. Cerdá X, Retana J, Cros S. 1998 Critical thermal limits in Mediterranean ant species: trade-off between mortality risk and foraging performance. *Funct. Ecol.* **12**, 45–55. (doi:10.1046/j.1365-2435.1998.00160.x)

130. Verberk WCEP, Bilton DT. 2015 Oxygen-limited thermal tolerance is seen in a plastron-breathing insect and can be induced in a bimodal gas exchanger. *J. Exp. Biol.* **218**, 2083–2088.

131. Le Lann C, Roux O, Serain N, Van Alphen JJM, Vernon P, Van Baaren J. 2011 Thermal tolerance of sympatric hymenopteran parasitoid species: does it match seasonal activity? *Physiol. Entomol.* **36**, 21–28. (doi:10.1111/j.1365-3032.2010.00758.x)

132. Kaspari M, Clay NA, Lucas J, Yanoviak SP, Kay A. 2014 Thermal adaptation generates a diversity of thermal limits in a rainforest ant community. *Glob. Change Biol.* **21**, 1092–1102. (doi:10.1111/gcb.12750)

133. Klok CJ, Chown SL. 2003 Resistance to temperature extremes in sub-Antarctic weevils: interspecific variation, population differentiation and acclimation. *Biol. J. Linn. Soc.* **78**, 401–414. (doi:10.1046/j.1095-8312.2003.00154.x)

134. Terblanche JS, Nyamukondiwa C, Kleynhans E. 2010 Thermal variability alters climatic stress resistance and plastic responses in a globally invasive pest, the Mediterranean fruit fly (*Ceratitis capitata*). *Entomol. Exp. Appl.* **137**, 304–315. (doi:10.1111/j.1570-7458.2010.01067.x)

135. Nyamukondiwa C, Chidawanyika F, Machekano H, Mutamiswa R, Sands B, Mgidiswa N, Wall R. 2018 Climate variability differentially impacts thermal fitness traits in three coprophagic beetle species. *PLOS ONE* **13**, e0198610. (doi:10.1371/journal.pone.0198610)

136. Hu PH, Appel AG. 2004 Seasonal Variation of Critical Thermal Limits and Temperature Tolerance in Formosan and Eastern Subterranean Termites (Isoptera: Rhinotermitidae). *Environ. Entomol.* **33**, 197–205. (doi:10.1603/0046-225X-33.2.197)

137. Vorhees AS, Gray EM, Bradley TJ. 2013 Thermal Resistance and Performance Correlate with Climate in Populations of a Widespread Mosquito. *Physiol. Biochem. Zool.* **86**, 73–81. (doi:10.1086/668851)

138. Calosi P, Bilton DT, Spicer JI, Votier SC, Atfield A. 2010 What determines a species’ geographical range? Thermal biology and latitudinal range size relationships in European diving beetles (Coleoptera: Dytiscidae). *J. Anim. Ecol.* **79**, 194–204.

139. Verberk WCEP, Calosi P, Spicer JI, Kehl S, Bilton DT. 2018 Does plasticity in thermal tolerance trade off with inherent tolerance? The influence of setal tracheal gills on thermal tolerance and its plasticity in a group of European diving beetles. *J. Insect Physiol.* **106**, 163–171. (doi:10.1016/j.jinsphys.2017.12.005)

140. Verberk WCEP, Sommer U, Davidson RL, Viant MR. 2013 Anaerobic Metabolism at Thermal Extremes: A Metabolomic Test of the Oxygen Limitation Hypothesis in an Aquatic Insect. *Integr. Comp. Biol.* **53**, 609–619. (doi:10.1093/icb/ict015)

141. Chown SL, Jumbam KR, Sørensen JG, Terblanche JS. 2009 Phenotypic variance, plasticity and heritability estimates of critical thermal limits depend on methodological context. *Funct. Ecol.* **23**, 133–140. (doi:10.1111/j.1365-2435.2008.01481.x)

142. Arribas P, Velasco J, Abellán P, Sánchez-Fernández D, Andújar C, Calosi P, Millán A, Ribera I, Bilton DT. 2012 Dispersal ability rather than ecological tolerance drives differences in range size between lentic and lotic water beetles (Coleoptera: Hydrophilidae). *J. Biogeogr.* **39**, 984–994. (doi:10.1111/j.1365-2699.2011.02641.x)

143. Verberk WC, Durance I, Vaughan IP, Ormerod SJ. 2016 Field and laboratory studies reveal interacting effects of stream oxygenation and warming on aquatic ectotherms. *Glob. Change Biol.* **22**, 1769–1778.

144. Terblanche JS, Klok CJ, Krafsur ES, Chown SL. 2006 Phenotypic plasticity and geographic variation in thermal tolerance and water loss of the tsetse *Glossina pallidipes* (Diptera: Glossinidae): implications for distribution modelling. *Am. J. Trop. Med. Hyg.* **74**, 786–794.

145. Slabber S, Chown SL. 2005 Differential responses of thermal tolerance to acclimation in the sub-Antarctic rove beetle *Halmaeusa atriceps*. *Physiol. Entomol.* **30**, 195–204. (doi:10.1111/j.1365-3032.2005.00448.x)

146. Garten CT, Gentry JB. 1976 Thermal Tolerance of Dragonfly Nymphs. II. Comparison of Nymphs from Control and Thermally Altered Environments. *Physiol. Zool.* **49**, 206–213.

147. Billman HG, Giersch JJ, Kappenman KM, Muhlfeld CC, Webb MAH. 2013 Thermal tolerance of meltwater stonefly *Lednia tumana* nymphs from an alpine stream in Waterton–Glacier International Peace Park, Montana, USA. *Freshw. Sci.* **32**, 597–605. (doi:10.1899/12-100.1)

148. Jumbam KR, Terblanche JS, Deere JA, Somers MJ, Chown SL. 2007 Critical thermal limits and their responses to acclimation in two sub-Antarctic spiders: *Myro kerguelenensis* and Prinerigone vagans. *Polar Biol.* **31**, 215. (doi:10.1007/s00300-007-0349-0)

149. Slatyer RA, Schoville SD. 2016 Physiological Limits along an Elevational Gradient in a Radiation of Montane Ground Beetles. *PLOS ONE* **11**, e0151959. (doi:10.1371/journal.pone.0151959)

150. Sánchez-Fernández D, Calosi P, Atfield A, Arribas P, Velasco J, Spicer JI, Millán A, Bilton DT. 2010 Reduced salinities compromise the thermal tolerance of hypersaline specialist diving beetles. *Physiol. Entomol.* **35**, 265–273. (doi:10.1111/j.1365-3032.2010.00734.x)

151. Shoup L, Houghton D. 2013 The Effect of Acclimation Temperature on the Critical Thermal Maximum of a Cold-water Population of *Pteronarcys dorsata* (Say) (Plecoptera: Pteronarcyidae). *Gt. Lakes Entomol.* **46**, 165–173.

152. Coccia C, Calosi P, Boyero L, Green AJ, Bilton DT. 2013 Does Ecophysiology Determine Invasion Success? A Comparison between the Invasive Boatman *Trichocorixa verticalis* *verticalis* and the Native *Sigara lateralis* (Hemiptera, Corixidae) in South-West Spain. *PLOS ONE* **8**, e63105. (doi:10.1371/journal.pone.0063105)

153. Clara Frasconi Wendt, Robin Verble-Pearson. 2016 Critical thermal maxima and body size positively correlate in red imported fire ants, *Solenopsis invicta*. *Southwest. Nat.* **61**, 79–83.

154. Diele-Viegas LM *et al.* 2018 Thermal physiology of Amazonian lizards (Reptilia: Squamata). *PloS One* **13**, e0192834.

155. van Berkum FH. 1988 Latitudinal Patterns of the Thermal Sensitivity of Sprint Speed in Lizards. *Am. Nat.* **132**, 327–343.

156. Williamson LU, Spotila JR, Standora EA. 1989 Growth selected temperature and CTM of young snapping turtles, *Chelydra serpentina*. *J. Therm. Biol.* **14**, 33–39. (doi:10.1016/0306-4565(89)90027-2)

157. Bennett AF, John-Alder H. 1986 Thermal Relations of Some Australian Skinks (Sauria: Scincidae). *Copeia* **1986**, 57–64. (doi:10.2307/1444888)

158. Li H, Zheng W, Mei W, Ji X. 2009 Temperature acclimation affects thermal reference and tolerance in three *Eremias* lizards (Lacertidae). *Curr. Zool.* **55**, 258–265.

159. Bauwens D, Garland T, Castilla AM, Van Damme R. 1995 Evolution of sprint speed in lacertid lizards: morphological, physiological, and behavioral covariation. *Evolution* **49**, 848–863.

160. Geng J, Dong W, Wu Q, Lu H-L. 2018 Thermal tolerance for two cohorts of a native and an invasive freshwater turtle species. *Acta Herpetol.* **13**, 83–88.

161. Caldwell AJ, While GM, Beeton NJ, Wapstra E. 2015 Potential for thermal tolerance to mediate climate change effects on three members of a cool temperate lizard genus, Niveoscincus. *J. Therm. Biol.* **52**, 14–23. (doi:10.1016/j.jtherbio.2015.05.002)

162. Li H, Qu Y, Ji X, Gao J, Xu X. 2015 Thermal preference, thermal tolerance and the thermal dependence of digestive performance in two *Phrynocephalus* lizards (Agamidae), with a review of species studied. *Curr. Zool.* **57**, 684–700. (doi:10.1093/czoolo/57.6.684)

163. Wang Z, Lu H, Ma L, Ji X. 2013 Differences in Thermal Preference and Tolerance among Three *Phrynocephalus* Lizards (Agamidae) with Different Body Sizes and Habitat Use. *Asian Herpetol. Res.* **4**, 214–220.

164. Lailvaux SP, Alexander GJ, Whiting MJ. 2003 Sex‐Based Differences and Similarities in Locomotor Performance, Thermal Preferences, and Escape Behaviour in the Lizard *Platysaurus intermedius wilhelmi*. *Physiol. Biochem. Zool. Ecol. Evol. Approaches* **76**, 511–521. (doi:10.1086/376423)

165. Du W-G, Yan S-J, Ji X. 2000 Selected body temperature, thermal tolerance and thermal dependence of food assimilation and locomotor performance in adult blue-tailed skinks, Eumeces elegans. *J. Therm. Biol.* **25**, 197–202. (doi:10.1016/S0306-4565(99)00022-4)

166. Youssef MK, Adolph SC, Richmond JQ. 2008 Evolutionarily conserved thermal biology across continents: The North American lizard Plestiodon gilberti (Scincidae) compared to Asian Plestiodon. *J. Therm. Biol.* **33**, 308–312. (doi:10.1016/j.jtherbio.2008.02.007)

167. Huang S, Huang S, Chen Y, Tu M. 2007 Thermal Tolerance and Altitudinal Distribution of Three *Trimeresurus* Snakes (Viperidae: Crotalinae) in Taiwan. *Zool. Stud.* **46**, 592–599.

168. Angilletta MJ, Hill T, Robson MA. 2002 Is physiological performance optimized by thermoregulatory behavior?: a case study of the eastern fence lizard, *Sceloporus undulatus*. *J. Therm. Biol.* **27**, 199–204. (doi:10.1016/S0306-4565(01)00084-5)

169. Huang S-P, Hsu Y, Tu M-C. 2006 Thermal tolerance and altitudinal distribution of two *Sphenomorphus* lizards in Taiwan. *J. Therm. Biol.* **31**, 378–385. (doi:10.1016/j.jtherbio.2005.11.032)

170. Ji X, Sun P-Y, Du W-G. 1996 Selected Body Temperature, Thermal Tolerance and Food Assimilation in a Viviparous Skink, *Sphenomorphus indicus*. *Neth. J. Zool.* **47**.

171. Huang S-P, Tu M-C. 2008 Cold Tolerance and Altitudinal Distribution of *Takydromus* Lizards in Taiwan. *Zool. Stud.***47**, 238–444.

172. Yang J, Sun Y-Y, An H, Ji X. 2008 Northern grass lizards (*Takydromus septentrionalis*) from different populations do not differ in thermal preference and thermal tolerance when acclimated under identical thermal conditions. *J. Comp. Physiol. B* **178**, 343–349. (doi:10.1007/s00360-007-0227-7)

173. Zhang Y-P, Ji X. 2004 The thermal dependence of food assimilation and locomotor performance in southern grass lizards, *Takydromus sexlineatus* (Lacertidae). *J. Therm. Biol.* **29**, 45–53. (doi:10.1016/j.jtherbio.2003.10.007)

174. Mautz WJ, Daniels CB, Bennett AF. 1992 Thermal Dependence of Locomotion and Aggression in a Xantusiid Lizard. *Herpetologica* **48**, 271–279.
